# Niacin Modulates SIRT1-Driven Signaling to Counteract Radiation-Induced Neurocognitive and Behavioral Impairments

**DOI:** 10.3390/ijms26115285

**Published:** 2025-05-30

**Authors:** Erdinç Tunç, Hatice Aygün, Mümin Alper Erdoğan, Yiğit Uyanıkgil, Oytun Erbaş

**Affiliations:** 1 Department of Anatomy, Faculty of Medicine, Biruni University, 34295 Istanbul, Türkiye; etunc@biruni.edu.tr; 2 Department of Physiology, Faculty of Medicine, Tokat Gaziosmanpaşa University, 60030 Tokat, Türkiye; 3 Department of Physiology, Faculty of Medicine, Izmir Katip Celebi University, 35620 Izmir, Türkiye; alpero86@gmail.com; 4 Department of Histology and Embryology, Faculty of Medicine, Ege University, 35040 Izmir, Türkiye; yigit.uyanikgil@ege.edu.tr; 5 BAMER, Faculty of Medicine, Biruni University, 34295 Istanbul, Türkiye; oytunerbas2012@gmail.com

**Keywords:** niacin, whole brain irradiation, oxidative stress, neuroinflammation, SIRT1, SIRT6, cognitive function, neuroprotection

## Abstract

Radiation exposure causes neuroinflammation, oxidative stress, and neuronal loss, leading to cognitive and behavioral impairments. This study aims to evaluate the effect of niacin interventions on whole-brain irradiation (WBI)-induced cognitive and behavioral impairment. Female Wistar rats were randomly assigned to Control (Group 1), Radiation +Saline (Group 2), and Radiation +niacin (Group 3) groups. Rats in the irradiated groups (Groups 2 and 3) received a single dose of 20 Gy photon irradiation. Group 2 received water seven days after irradiation, while Group 3 received niacin (60 mg/kg, 2 mL) oral gavage for 15 days. On days 22, 23, and 24, behavioral assessments were performed, including the Open Field Test, the Sociability Test, and the Passive Avoidance Learning (PAL) task. Biochemical analyses included MDA, BDNF, TNF-α, CREB), SIRT1, and SIRT6 measured by ELISA. Histological assessments included neuronal density and GFAP immunostaining in CA1 and CA3 regions of the hippocampus and cerebellar Purkinje neurons. Radiation exposure importantly increased MDA and TNF-α levels, while SIRT1, SIRT6, BDNF, and CREB were notably reduced. This was accompanied by neuronal loss in the cerebellum and hippocampus, astrogliosis, and behavioral and cognitive deficits. Niacin treatment significantly decreased MDA and TNF-α levels while increasing BDNF, CREB, SIRT1, and SIRT6 expression, attenuating neuronal apoptosis. Immunohistochemical analysis demonstrated that niacin treatment enhanced neuronal density in the CA1 and CA3 regions of the hippocampus and cerebellar Purkinje neurons while reducing GFAP immunoreactivity in the CA1, CA3, and cerebellum following WBI. Behaviorally, niacin treatment improved social interaction, locomotor activity, and memory performance, underscoring its neuroprotective potential against WBI-induced damage. These findings suggest that niacin may ameliorate behavioral and cognitive impairments following whole brain irradiation by activating the SIRT1/CREB/BDNF or SIRT1/SIRT6/MDA/TNF-α signaling pathway.

## 1. Introduction

Radiotherapy is a key treatment for brain tumors, including glioma, glioblastoma, and metastases [1]. Although therapeutic irradiation effectively targets tumor cells, it also induces collateral damage to normal brain cells. Radiation-induced brain injury is commonly observed following fractionated partial or whole-brain irradiation (WBI), presenting a syndrome characterized by both functional and anatomical deficits [2].

Exposure to radiation is linked to various side effects, including cognitive impairment, depression, anxiety, and altered social interaction [3]. Radiation-induced cognitive dysfunction affects up to 90% of patients with brain tumors who survive beyond six months after WBI [4,5]. Additionally, A clinical study reported that 17% of brain tumor patients experience depression, while 30% suffer from anxiety [6]. An experimental study noted that mice receiving a low dose of cosmic radiation displayed increased anxiety, diminished social interaction, and impaired recognition memory [7]. The behavioral outcomes of radiation injury thus encompass a spectrum from heightened anxiety and exploratory deficits to severe learning and memory impairments, all of which significantly diminish quality of life.

The brain is recognized as particularly vulnerable to oxidative damage, and the susceptibility of neuronal membranes to peroxidation reinforces this perspective. Brain tissue, despite its relatively small mass (2%), accounts for a disproportionately large share (20%) of total oxygen consumption. While it is enriched in highly peroxidizable fatty acids, it is not particularly endowed with antioxidant defenses. It is well established that lipid-rich environments are more susceptible to free radical-induced damage [8,9]. WBI-induced brain tissue damage is primarily driven by oxidative stress, leading to lipid peroxidation, mitochondrial dysfunction, and DNA damage [10]. Moreover, activated microglia exacerbate neuroinflammation and neuronal loss by secreting proinflammatory cytokines (i.e., TNF-α) [2,11]. Studies have shown that inflammation and oxidative stress are important in radiation-induced behavioral changes and cognitive impairment. Studies indicated that niacin (vitamin B3) has been proven to have anti-inflammatory and antioxidant properties [12]. A review of the existing studies reveals that there is no research investigating the efficacy of niacin in the context of whole-brain irradiation.

Therefore, this study aimed to evaluate the neuroprotective potential of niacin in a rat model of whole-brain irradiation using behavioral, biochemical, and histopathological assessments. Behavioral tests included the Open Field Test (for locomotion and anxiety), the Three-Chamber Sociability Test (for social interaction), and the Passive Avoidance Learning Test (for memory performance). Histological evaluation focused on GFAP immunostaining and neuronal density in the hippocampus and cerebellum to assess neuroinflammation and neuronal loss.

We hypothesized that niacin supplementation may alleviate WBI-induced behavioral and cognitive impairments by reducing oxidative stress and inflammation through modulation of the SIRT1/CREB/BDNF and SIRT1/SIRT6 signaling pathways.

## 2. Results

This study demonstrated significant alterations in oxidative stress, neuroinflammatory markers, neuronal integrity, and behavioral outcomes following radiation exposure, which were significantly attenuated by niacin administration.

No data points were excluded from the analysis. Visual inspection and z-score thresholds (±2 SD) were used to detect potential outliers, but no values met the exclusion criteria.

### 2.1. Oxidative Stress and Inflammation

#### 2.1.1. MDA Analysis

**Figure 1:**
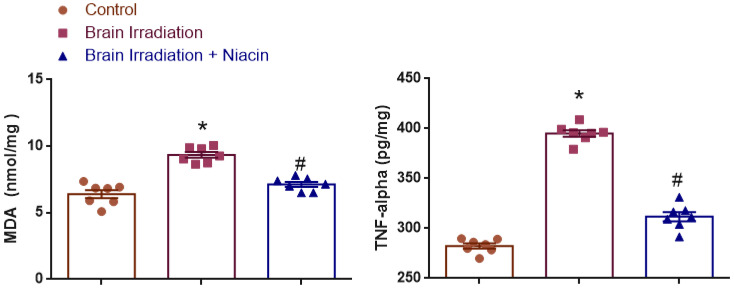
Effects of brain irradiation and niacin treatment on MDA and TNF-α levels. Data are presented as mean ± SEM. * *p* < 0.001 compared to the control group; # *p* < 0.001 compared to the brain irradiation group.

To assess lipid peroxidation, MDA levels were statistically analyzed. Normality was confirmed (Shapiro–Wilk: *p* > 0.05), and variances were homogeneous (Levene’s test: F(2,18) = 1.907, *p* = 0.177). One-way ANOVA showed a significant treatment effect (F(2,18) = 40.339, *p* < 0.0001). MDA levels were significantly higher in the irradiation group than in the control (*p* < 0.001), and significantly lower in the irradiation +niacin group compared to the irradiation group (*p* < 0.001) (Figure 1).

#### 2.1.2. TNF-α Analysis

TNF-α levels were evaluated to examine neuroinflammatory responses. Shapiro–Wilk test confirmed normality (*p* > 0.05), and Levene’s test supported variance homogeneity (F(2,18) = 0.583, *p* = 0.569). ANOVA revealed a robust treatment effect (F(2,18) = 255.314, *p* < 0.0001). TNF-α was significantly elevated in the irradiation group vs. the control (*p* < 0.001) and significantly attenuated in the irradiation +niacin group compared to the irradiation group (*p* < 0.001) (Figure 1).

### 2.2. Neurotrophic and Synaptic Plasticity Markers

#### 2.2.1. BDNF Analysis

**Figure 2:**
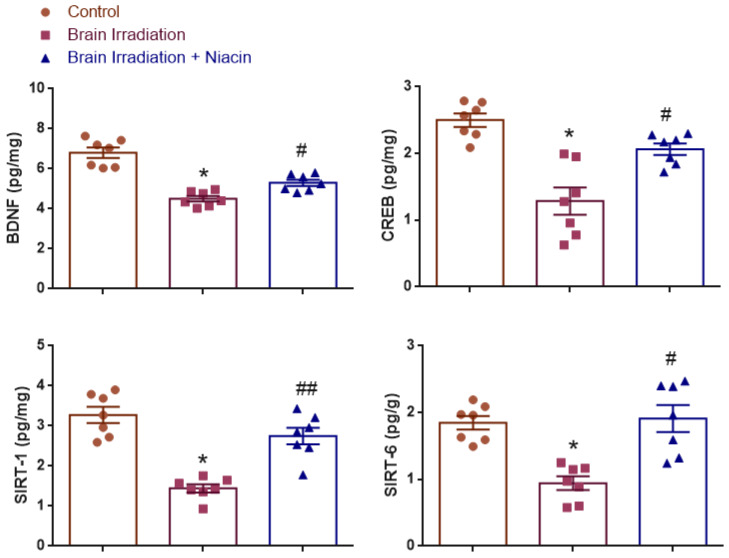
Effects of brain irradiation and niacin treatment on neurotrophic and neuroplasticity-related markers (BDNF, CREB, SIRT-1, and SIRT-6) in rat brain tissue. Data are expressed as mean ± SEM (*n* = 7 per group). * *p* < 0.001 vs. control group; # *p* < 0.01, ## *p* < 0.001 vs. brain irradiation group.

BDNF levels were analyzed to assess neurotrophic support. Normality was satisfied (Shapiro–Wilk: *p* > 0.05); however, Levene’s test indicated heterogeneity of variances (F(2,18) = 5.716, *p* = 0.012). One-way ANOVA with Tamhane’s T2 post hoc test revealed a significant group effect (F(2,18) = 37.21, *p* < 0.0001). BDNF levels were significantly reduced in the irradiation group vs. the control (*p* < 0.001) and significantly elevated in the irradiation +niacin group compared to the irradiation group (*p* = 0.007) (Figure 2).

#### 2.2.2. CREB Analysis

CREB levels were assessed as a marker of neuroplasticity. Data met parametric assumptions (Shapiro–Wilk: *p* > 0.05; Levene’s test: F(2,18) = 3.374, *p* = 0.057). One-way ANOVA showed a significant treatment effect (F(2,18) = 19.205, *p* < 0.0001). CREB levels were significantly lower in the irradiation group than in the control (*p* < 0.001) and significantly higher in the irradiation +niacin group compared to the irradiation group (*p* = 0.003) (Figure 2).

#### 2.2.3. SIRT1 Analysis

SIRT1 levels were analyzed to evaluate neuroprotective effects. Normality and homogeneity of variances were confirmed (Shapiro–Wilk: *p* > 0.05; Levene’s test: F(2,18) = 2.373, *p* = 0.122). One-way ANOVA revealed a significant effect (F(2,18) = 28.43, *p* < 0.001). SIRT1 expression was significantly reduced in the irradiation group compared to the control (*p* < 0.001), but significantly increased in the irradiation +niacin group compared to the irradiation group (*p* < 0.001) (Figure 2).

#### 2.2.4. SIRT6 Analysis

SIRT6 levels were examined for treatment effects. Normality was confirmed (Shapiro–Wilk: *p* > 0.05), while Levene’s test showed variance heterogeneity (F(2,18) = 5.023, *p* = 0.018). Therefore, ANOVA followed by Tamhane’s T2 test was used. A significant effect was found (F(2,18) = 14.279, *p* < 0.001), with SIRT6 levels lower in the irradiation group than in the control (*p* < 0.001) and significantly higher in the irradiation +niacin group than in the irradiation group (*p* = 0.006) (Figure 2).

### 2.3. Behavioral Assessments

Behavioral evaluations included sociability, open field, and passive avoidance tests. For each behavioral outcome, a post hoc power analysis was conducted using G*Power (version 3.1.9.7). The analyses revealed large to very large observed effect sizes across tests (Cohen’s *f* range: 0.97–2.01), with actual power values between 0.96 and 0.99. These results confirm that the sample size (*n* = 7 per group) was statistically sufficient to detect meaningful group differences with high confidence (α = 0.05, three groups).

#### 2.3.1. Sociability Test

##### Time Spent (%) with Stranger Rat

**Figure 3:**
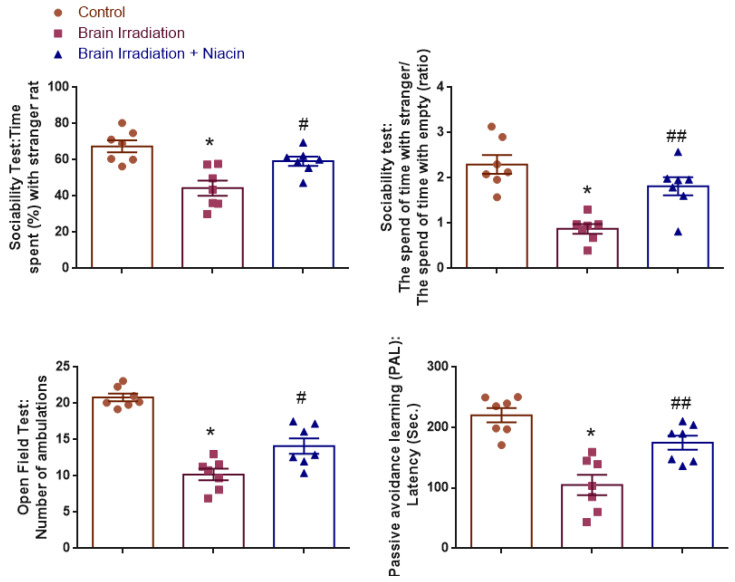
Behavioral outcomes following brain irradiation and niacin treatment. Graphs show group comparisons for sociability (time spent with stranger rat and sociability ratio), locomotor activity (ambulations in the open field), and memory performance (step-through latency in the passive avoidance test). Data are presented as mean ± SEM.* *p* < 0.001 vs. control; # *p* < 0.05, ## *p* < 0.01 vs. brain irradiation group.

Time spent with the stranger rat was analyzed to assess social preference. Normality was confirmed for all groups (Shapiro–Wilk: *p* > 0.05), and variance homogeneity was verified (Levene’s test: F(2,18) = 1.80, *p* = 0.194). ANOVA revealed a significant group effect (F(2,18) = 11.82, *p* = 0.001). Post hoc analysis showed that irradiated rats spent significantly less time with the stranger rat than controls (*p* < 0.001), while niacin-treated rats spent significantly more time than the irradiation group (*p* = 0.017) (Figure 3).

##### The Time Spent with Strangers/the Time Spent Alone (Ratio)

The ratio of time spent with the stranger versus the empty chamber was used to quantify sociability. Data were normally distributed (Shapiro–Wilk: *p* > 0.05) and variances were homogeneous (Levene’s test: F(2,18) = 0.96, *p* = 0.403). A significant group effect was observed (F(2,18) = 16.69, *p* < 0.001). Sociability ratios were significantly lower in the irradiation group compared to the control (*p* < 0.001) and significantly higher in the irradiation +niacin group compared to the irradiation group (*p* = 0.004) (Figure 3).

#### 2.3.2. Open Field Test (OFT)

Ambulation counts were analyzed to evaluate locomotor activity. Normality was confirmed (Shapiro–Wilk: *p* > 0.05), and homogeneity of variances was supported (Levene’s test: F(2,18) = 3.39, *p* = 0.056). ANOVA showed a strong group effect (F(2,18) = 42.30, *p* < 0.001). Irradiated rats exhibited reduced ambulation relative to controls (*p* < 0.001), while the niacin-treated group showed significant improvement compared to the irradiation group (*p* = 0.010) (Figure 3).

#### 2.3.3. Passive Avoidance Learning (PAL)

Latency to enter the dark compartment was analyzed to evaluate memory retention. Data met normality (Shapiro–Wilk: *p* > 0.05) and variance homogeneity (Levene’s test: F(2,18) = 1.02, *p* = 0.381). ANOVA identified a significant group effect (F(2,18) = 18.01, *p* < 0.001). Latency was significantly shorter in irradiated rats compared to controls (*p* < 0.001), while the niacin group exhibited longer latencies than the irradiation group (*p* = 0.005) (Figure 3).

### 2.4. Histological and Immunohistochemical Findings

#### 2.4.1. Neuronal Count in CA1 Region

To assess neuronal integrity in the hippocampal CA1 region, neuron counts were statistically analyzed. Normality was confirmed for all groups (Shapiro–Wilk: *p* > 0.05), and Levene’s test indicated homogeneity of variances (F(2,18) = 1.512, *p* = 0.247).

**Figure 4:**
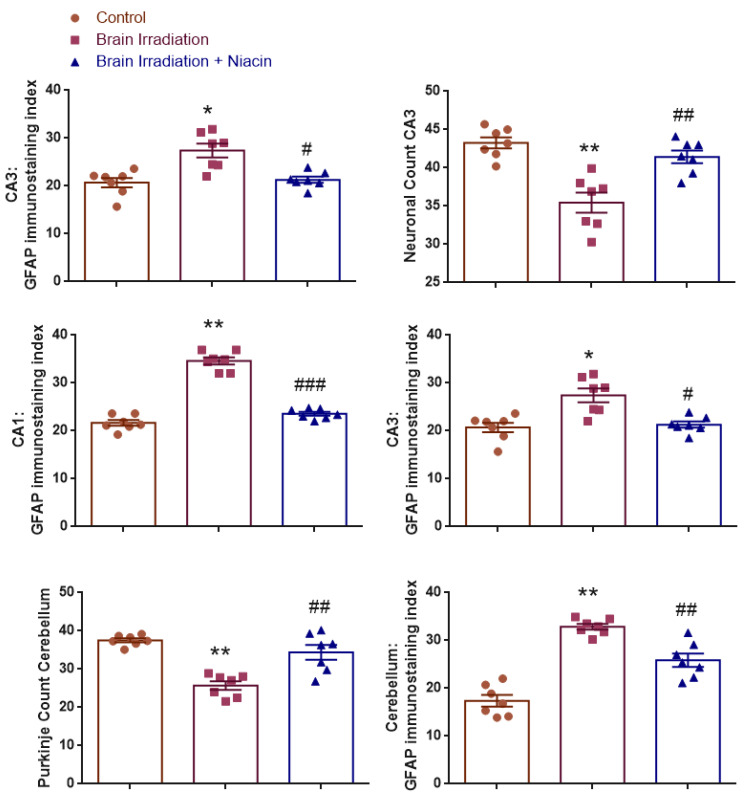
Histological and immunohistochemical evaluations of neuronal survival (CA1, CA3, and Purkinje cell counts) and astrocytic activation (GFAP immunostaining index in CA1, CA3, and cerebellum) across groups. Brain irradiation significantly reduced neuronal counts and increased GFAP expression, indicating neurodegeneration and reactive gliosis. Niacin treatment partially restored neuronal integrity and attenuated astrocyte activation. * *p* < 0.01, ** *p* < 0.001 distinct from control groups; # *p* < 0.05, ## *p* < 0.01, ### *p* < 0.001 distinct from Brain Irradiation group.

**Figure 5:**
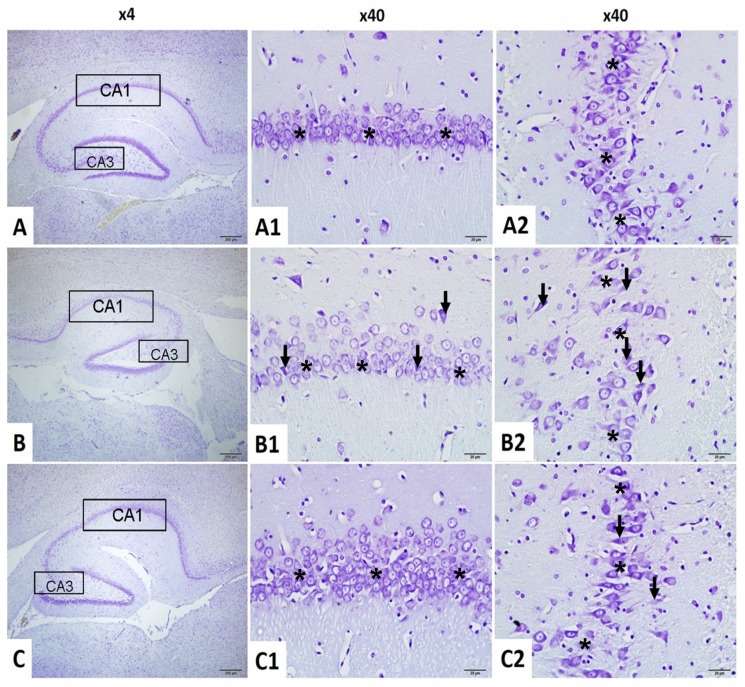
CA1 and CA3 regions of hippocampus Cresyl violet stain ×4 and ×40 magnification. (**A**–**A2**): Normal Control Group female Rats CA1 and CA3 have normal pyramidal neuron (asterisk); (**B**–**B2**): Brain Irradiation and saline group female rats have decreased Normal pyramidal neuron count (asterisk) and increased dysmorphological changes pyramidal neuron (arrow); (**C**–**C2**): Brain Irradiation and Niacin group female rats have have increased Normal pyramidal Neuron count (asterisk), improved pyramidal neuron morphology changes (asterisk) and decreased dysmorphological changes pyramidal Neuron (arrow). (Scale bars for 1 cm = 50 μm).

One-way ANOVA revealed a significant group effect (F(2,18) = 58.249, *p* < 0.001). Neuronal counts were significantly reduced in the brain irradiation group compared to the control (*p* < 0.001). The irradiation +niacin group demonstrated significantly higher counts than the irradiation group (*p* < 0.001), indicating a neuroprotective effect of niacin (Figure 4 and Figure 5).

#### 2.4.2. Neuronal Count in CA3 Region

Neuron counts in the hippocampal CA3 area were analyzed. Normality and homogeneity of variances were satisfied (Shapiro–Wilk: all *p* > 0.05; Levene’s test: F(2,18) = 2.949, *p* = 0.078).

ANOVA indicated a significant group effect (F(2,18) = 17.093, *p* < 0.001). Post hoc analysis showed a significant reduction in the irradiation group compared to the control (*p* < 0.001) and a significant increase in the irradiation +niacin group compared to the irradiation group (*p* = 0.002, *p* < 0.01). This suggests a protective effect against radiation-induced neurodegeneration (Figure 4 and Figure 5).

#### 2.4.3. CA1–GFAP Immunostaining Index

Astrocyte activation in the CA1 region was evaluated by GFAP immunostaining. Normality and homogeneity were confirmed (Shapiro–Wilk: all *p* > 0.05; Levene’s test: F(2,18) = 0.997, *p* = 0.388).

**Figure 6:**
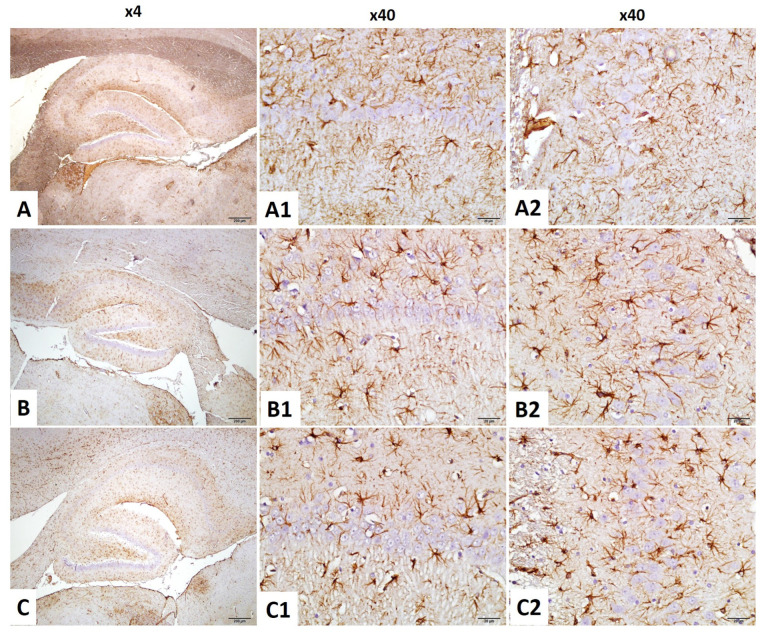
CA1 and CA3 of hippocampus ×40 magnification. Astrogliosis was characterized by GFAP immunostaining (Brown staining). (**A**–**A2**) Normal Control Group female Rats CA1 and CA3, (**B**–**B2**) Brain Irradiation and saline group female rats have increased glial activity CA1 and CA3. (**C**–**C2**) Brain Irradiation and Niacin group female rats have decreased glial activity in CA1 and CA3 (Scale bars for 1 cm = 50 μm).

A significant group effect was found (F(2,18) = 134.456, *p* < 0.001). GFAP expression was significantly increased in the irradiation group compared to the control (*p* < 0.001), suggesting reactive gliosis. The irradiation +niacin group showed significantly reduced GFAP levels compared to the irradiation group (*p* < 0.001), indicating attenuation of astrogliosis (Figure 4 and Figure 6).

#### 2.4.4. CA3–GFAP Immunostaining Index

GFAP immunoreactivity in the CA3 region was analyzed. Normality was confirmed; however, Levene’s test indicated a violation of variance homogeneity (F(2,18) = 3.880, *p* = 0.040). Therefore, Tamhane’s T2 post hoc test was used.

ANOVA revealed a significant group effect (F(2,18) = 11.990, *p* < 0.001). GFAP levels were significantly elevated in the irradiation group compared to the control (*p* = 0.008, *p* < 0.01), suggesting reactive gliosis. The irradiation +niacin group exhibited significantly lower levels compared to the irradiation group (*p* = 0.013, *p* < 0.05), suggesting attenuation of astrocytic activation (Figure 4 and Figure 6).

#### 2.4.5. Purkinje Cell Count in the Cerebellum

To assess cerebellar neuronal integrity, Purkinje cell numbers were analyzed. While normality was confirmed, Levene’s test revealed unequal variances (F(2,18) = 8.904, *p* = 0.002), and thus Tamhane’s T2 post hoc test was used.

**Figure 7:**
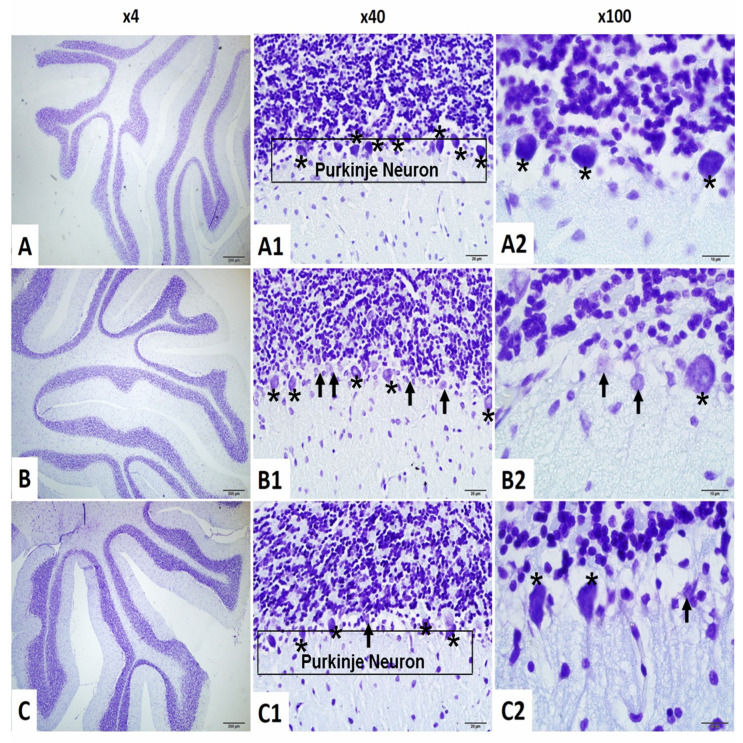
Cerebellum Cresyl violet stain ×40 and ×100 magnification. (**A**–**A2**), Normal Control Group female Rats cerebellum have normal Purkinje Neuron (asterisk); (**B**–**B2**): Brain Irradiation and saline group female rats have decreased Normal Purkinje Neuron count (asterisk) and increased dysmorphological changes Purkinje Neuron (arrow); (**C**–**C2**): Brain Irradiation and Niacin group female rats have increased Normal Purkinje Neuron count (asterisk), improved Purkinje neuron morphology changes (asterisk) and decreased dysmorphological changes Purkinje Neuron (arrow). (Scale bars for 1 cm = 50 μm).

A significant group effect was observed (F(2,18) = 21.907, *p* < 0.001). Purkinje cell counts were significantly reduced in the irradiation group compared to the control (*p* < 0.001). The irradiation +niacin group had significantly higher counts than the irradiation group (*p* = 0.009, *p* < 0.01), suggesting a potential role in the maintenance of cerebellar function (Figure 4 and Figure 7).

#### 2.4.6. GFAP Immunostaining Index in the Cerebellum

Astrocyte activity in the cerebellum was measured via GFAP staining. Both normality and homogeneity were confirmed (Shapiro–Wilk: all *p* > 0.05; Levene’s test: F(2,18) = 2.597, *p* = 0.102).

**Figure 8:**
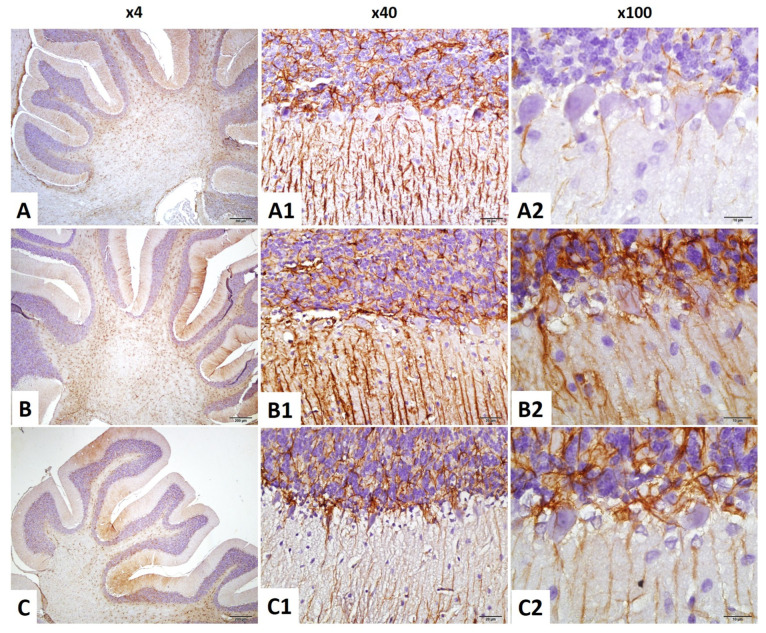
Female rats’ cerebellum ×40 and ×100 magnification. GFAP staining (brown) revealed astrogliosis. (**A**–**A2**) Control rats; (**B**–**B2**) Brain Irradiation rats have enhanced glial level; (**C**–**C2**) Brain Irradiation +niacin rats have reduced glial activity. Scale bars: 50 μm for ×40 magnification images (**A1**,**A2**,**B1**,**B2**,**C1**,**C2**).

ANOVA revealed a significant group effect (F(2,18) = 47.108, *p* < 0.001). GFAP expression significantly increased in the irradiation group compared to control (*p* < 0.001), whereas the irradiation +niacin group exhibited a significant decrease compared to the irradiation group (*p* = 0.001, *p* < 0.01), reflecting anti-gliotic effects of niacin (Figure 4 and Figure 8).

## 3. Discussion

This study demonstrated that niacin effectively attenuates whole brain irradiation (WBI)-induced oxidative stress, neuroinflammation, neuronal loss, and behavioral deficits. The improvements in neuronal survival, synaptic plasticity markers, and behavior suggest that niacin’s neuroprotective effects are mediated through the modulation of oxidative stress, neuroinflammation, and the SIRT1/CREB/BDNF or SIRT1/SIRT6/TNF-α/MDA pathway.

Cranial irradiation is commonly used to treat brain tumors; yet its potential to cause delayed cognitive and emotional dysfunction raises concerns about its impact on patients’ quality of life [13,14]. Additionally, previous experimental studies reported that exposure to radiation led to increased anxiety, reduced social interaction, and impaired recognition memory in male and female animals [7,15,16]. Previous in vitro studies have demonstrated radiation-induced brain dysfunction; a single 20 Gy dose impairs hippocampal-dependent behavior by disrupting neurogenesis and neural plasticity-related signaling [15,16]. Similarly, the present study observed cognitive deficits and behavioral changes following 20 Gy WBI.

In this study, a shortened latency time in the passive avoidance test demonstrated WBI-induced cognitive decline. At the same time, radiation-induced anxiety was evidenced by reduced locomotor activity in the open field test. Furthermore, radiation-exposed animals exhibited significantly less interaction with novel conspecifics in the sociability test, indicating impaired sociability. These findings align with previous experimental studies on WBI [15,17,18].

These behavioral and cognitive outcomes were observed alongside biochemical alterations in the brain and pronounced neurodegenerative histopathological modifications in the hippocampal tissues. Moreover, histopathological analysis confirmed WBI-induced neurodegeneration and showed an important decrease in the percentage of neurons in hippocampal CA1 and CA3 regions and a marked decrease in the percentage of Purkinje cells in the cerebellum.

It has been proven in many studies that the CA3 and CA1 regions of the hippocampus have a critical role in spatial memory and memory retrieval, while the cerebellum plays a role in motor learning and coordination-based memory processes [19]. Studies indicated that WBI caused significant neuronal loss in CA3 and CA1 [20]. Very few studies have investigated the effects of whole-brain irradiation on the cerebellum. One study reported a significant decrease in the number of Purkinje cells after WBI [21]. In the present study, the number of neurons in the cerebellum and CA1/CA3 regions of the hippocampus decreased after WBI. Niacin supplementation reversed this situation.

Conversely, niacin treatment in irradiated rats effectively reversed behavioral and cognitive impairments and preserved the standard histological architecture of hippocampal and cerebellar tissues. Additionally, niacin administration increased the percentage of intact neurons and promoted neuronal regeneration. These findings align with previous studies highlighting neuroprotective effects of niacin in various experimental models of neurodegenerative diseases and clinical studies [12,22].

From a pathophysiological perspective, multiple mechanisms have been implicated in developing WBI-induced neurotoxicity.

Indeed, oxidative stress and neuroinflammation are the most significant neurotoxic stimuli that trigger a series of signaling pathways, leading to cognitive dysfunction and behavioral changes [11,23] Numerous studies have provided clear evidence of oxidative stress as a key factor in radiation-induced brain damage [24,25,26]. ROS-induced oxidative stress leads to membrane integrity disruption and DNA damage [26]. MDA is a marker of lipid peroxidation. It is considered a key indicator of cell membrane damage [11]. In the present study, we have shown that niacin alleviated oxidative stress after WBI by reducing the level of MDA, suggesting that niacin has antioxidant properties.

Moreover, TNF-α, an important pro-inflammatory cytokine, plays a crucial role in WBI-induced neuroinflammation [15,27]. Upregulation of TNF-α leads to microglial overactivation [27]. On the one hand, microglial activation triggers TNF-α release and generates ROS, creating a positive feedback loop that leads to disruption of the blood-brain barrier and exacerbates neuronal death [27]. In the present study, increased TNF-α levels in the brain after WBI decreased after niacin supplementation. Furthermore, activated microglia are known to play an important role in radiation-induced brain gliosis by causing hypertrophy of astrocytic processes. The GFAP is an important marker of astrocytic activation and neuroinflammation [27]. Several studies have shown that single-dose irradiation increases GFAP expression in astrocytes [15,28]. One study reported that increased activation of GFAP in the hippocampus impaired synaptic transmission from CA3 to CA1 and impaired retrieval of remote memory [29]. This study indicated that niacin decreased the GFAP levels in the CA1 and CA3 regions of the hippocampus and cerebellum.

In addition, WBI also significantly affects neurotrophic signaling. BDNF is a protein regulating cognitive function [30]. BDNF activates CREB, which upregulates BDNF expression and creates a positive feedback loop that promotes memory formation [31]. Studies have reported that radiation exposure suppresses the CREB/BDNF signaling pathway, contributing to emotional disturbances and cognitive impairments [16,17,32]. This study demonstrated that niacin plays an important role in the prevention of WBI-induced behavioral and cognitive impairment by activating the CREB/BDNF signaling pathway.

The silent information regulator (sirtuin/SIRT) is a nicotinamide adenine dinucleotide (NAD^+^)-dependent protein deacetylase [33]. SIRT1- and SIRT6-mediated deacetylation play a critical role in regulating inflammation, oxidative stress, and apoptosis, thereby being essential for maintaining cellular homeostasis [33,34,35,36]. A recent study reported that WBI induced a decrease in SIRT1 expression in the rat hippocampus, which was associated with cognitive impairment [16]. However, no study has examined the activation of SIRT6 in rats after WBI.

Numerous studies showed the anti-inflammatory and antioxidant properties of SIRT1 and SIRT6, primarily through reducing MDA and TNF-α levels [33,35]. SIRT1 has been shown to inhibit TNF-α-mediated inflammation via NF-κB suppression [37,38] and attenuate pro-inflammatory cytokine expression in endothelial and macrophage cells [39,40]. Similarly, many studies have reported that SIRT6 suppresses TNF-α in a neurodegenerative disease model [41,42]. Furthermore, several studies indicated that SIRT1 activation upregulates antioxidant enzymes such as SOD and CAT, counteracting oxidative stress and inflammation in neural and vascular tissues [43,44,45]. Niacin-induced SIRT1 activation enhances NAD^+^ levels, reduces TNF-α, and mitigates oxidative stress [46,47].

Furthermore, studies have shown that SIRT1 activation has an effect on the CREP/BDNF pathway [48,49]. A previous study showed that SIRT1 acts as a positive modulator of the Akt/CREB/BDNF pathway and thus contributes to the improvement of cognitive functions and regulation of hippocampal neurogenesis [49].

SIRT1 and SIRT6 are an NAD +dependent deacetylase [36]. Many studies indicated that niacin can increase the activity of SIRT1 and SIRT6 [36,46,47]. Based on studies and findings, niacin supplementation may improve behavioral and cognitive functions after WBI by increasing SIRT1/SIRT6 activation in the brains of irritated rats. These results suggest that niacin may have some potential in the treatment of behavioral and cognitive disorders following WBI.

In addition to direct neuronal and glial injury, whole-brain irradiation (WBI) induces vascular damage characterized by microvascular rarefaction, endothelial cell senescence, and blood–brain barrier (BBB) dysfunction [50,51,52]. These changes impair neurovascular coupling and cerebral perfusion, while BBB disruption exacerbates neuroinflammatory cascades [53,54]. SIRT1, an NAD^+^-dependent deacetylase, plays a key role in maintaining endothelial integrity by upregulating tight junction proteins and suppressing oxidative stress and inflammation [55]. Our findings support this hypothesis, as niacin restored SIRT1 and SIRT6 protein levels and reduced TNF-α and MDA levels, suggesting a potential contribution of these sirtuins to the observed neuroprotection. Overall, by mitigating microvascular injury and preserving endothelial health through SIRT1/SIRT6-associated pathways, niacin may help maintain BBB integrity and cerebral perfusion following WBI, complementing its direct antioxidative and anti-inflammatory effects. Future studies assessing microvascular structure and function, as well as direct measurement of sirtuin activity, are warranted to clarify these mechanistic pathways and enhance the translational potential of niacin in radiation-induced brain injury [54,56].

## 4. Materials and Methods

### 4.1. Animals

**Figure 9:**
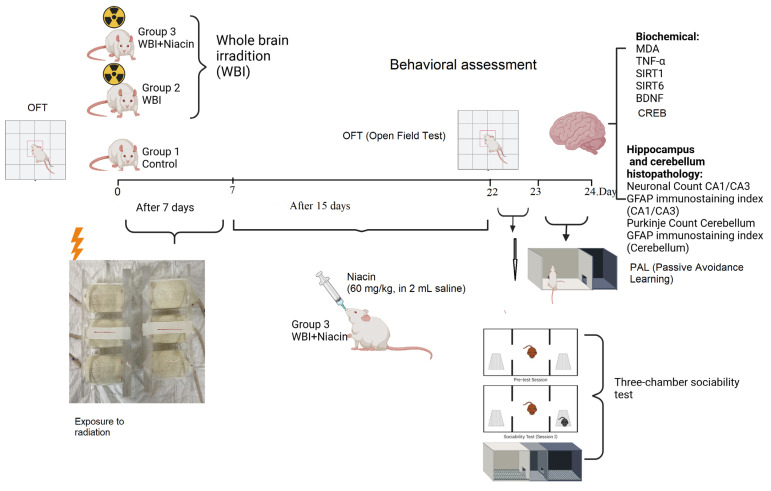
Experimental procedure and timeline.

Twenty-one adult female Wistar albino rats (body weight 150–200 g/10–12 weeks) obtained from Bilim University Experimental Animal Laboratory were used. The rats were housed at a temperature of 22 ± 2 °C, 12 light/12 dark cycle, and appropriate humidity, in accordance with standard laboratory animal conditions. The study was approved by Bilim University/Animal Ethics Committee (number: 2725022302) (Figure 9).

### 4.2. Experimental Procedures

Twenty-one adult female Wistar albino rats (10–12 weeks old, 150–200 g) were randomly assigned into three groups (*n* = 7 per group):

1. Group 1 (Control): Rats that were not exposed to whole-brain irradiation and were maintained under standard laboratory conditions. These animals received 2 mL of saline via oral gavage once daily for 15 consecutive days.
2. Group 2 (WBI +Saline): Rats that underwent whole-brain irradiation and subsequently received 2 mL of saline via oral gavage once daily for 15 consecutive days, beginning on Day 7 post-irradiation.
3. Group 3 (WBI +Niacin): Rats exposed to whole-brain irradiation and treated with niacin (60 mg/kg/day, dissolved in 2 mL sterile 0.9% NaCl) via oral gavage for 15 consecutive days, starting on Day 7 post-irradiation.

To ensure procedural consistency across groups, all animals—including those in the control group—received oral gavage once daily for 15 days. The same procedure and volume (2 mL) were applied across all groups, with niacin dissolved in saline only in the treatment group. All animals had free access to standard chow and drinking water ad libitum throughout the experimental period.

Prior to treatment, all animals underwent a 5-min baseline open field test to evaluate spontaneous locomotor activity. On Day 0, rats in Groups 2 and 3 received a single 20 Gy dose of cranial irradiation. Irradiation was performed under light general anesthesia using ketamine (50 mg/kg) and xylazine (10 mg/kg) to minimize movement during the procedure. Group 1 received no irradiation. From Days 7 to 21, treatment protocols were applied according to group assignments. Behavioral testing was conducted on Days 22–24 in the following order: Open Field Test (locomotor activity and anxiety-like behavior), Three-Chamber Sociability Test (social interaction), and Passive Avoidance Learning Test (memory performance).

After completion of behavioral assessments, all animals were anesthetized with ketamine (90 mg/kg) and xylazine (10 mg/kg) to achieve a deep surgical plane and euthanized by decapitation using a guillotine. Brains were rapidly extracted within 1–2 min postmortem and sagittally bisected.

The right hemisphere was immediately frozen at –20 °C for biochemical analyses, including measurements of BDNF, CREB, TNF-α, SIRT-1, and SIRT-6 using rat-specific ELISA kits. The left hemisphere was fixed in 10% formaldehyde prepared in 0.1 M phosphate-buffered saline (PBS) for histopathological evaluation. Histological analyses included neuronal counts in the CA1 and CA3 regions of the hippocampus, GFAP immunostaining indices in both regions, and Purkinje cell counts and GFAP immunoreactivity in the cerebellum.

### 4.3. Irradiation Procedure

Prior to irradiation, simulation was performed using a CT simulator (GE BrightSpeed, GE Healthcare, Chicago, IL, USA) with 1 mm slice thickness. The acquired images were transferred to the Eclipse treatment planning system (version 8.9, Varian Medical Systems, Palo Alto, CA, USA) for dose calculation. Further technical parameters of the irradiation plan (e.g., use of multileaf collimators, dose calculation algorithm, and animal immobilization method) were based on previously established protocols by our collaborating team [57].

Whole-brain irradiation (WBI) was performed under light general anesthesia using ketamine (50 mg/kg) and xylazine (10 mg/kg) to minimize movement. A single dose of 20 Gy was delivered using 6 MV photon beams, with a 1 cm bolus placed over the cranium to ensure uniform dose distribution to the brain surface. The rats were positioned in a prone position, and irradiation was applied at a rate of 1 Gy/min using the source-axis distance (SAD) technique. Following the procedure, animals were returned to their housing environment and monitored until full recovery [57].

### 4.4. Behavioral Tests

#### 4.4.1. Three-Chamber Sociability Test

The sociability test was conducted as previously defined [57]. Plexiglas cage (40 × 90 × 40 cm) with an open top divided into 3 equal chambers (40 × 30 × 40 cm) by 2 walls was used to evaluate social interaction.

On day 1, experimental rats were habituated to the test apparatus for 5 min (pre-test session). After 24 h, the Stranger 1 rat was placed in a small plastic cage in a side compartment and an empty plastic cage in the third space to test sociality. The test female rat was then placed in the central chamber and considered to be in the chamber when its two front paws and feet entered the cage, and the time spent in each cage area was monitored by camera for ten minutes (session I). To remove odor cues, the test area was cleaned with alcohol after each rat test. The percentage of time spent with strangers and the percentage of time spent in empty space were evaluated [58].

The stranger rats used in the tests were female Wistar albino rats of similar age and weight that had not previously been exposed to the experimental animals. To prevent habituation effects and maintain social novelty, a different stranger rat was used in each testing session. These rats were housed separately under standard conditions and were only introduced into the testing apparatus during the behavioral sessions.

#### 4.4.2. Open-Field Test (OFT)

The Open Field Test (OFT) is a widely used behavioral paradigm to evaluate locomotor activity and anxiety-like behavior in rodents [59,60,61]. Prior to the experimental procedures, all animals underwent a 5-min baseline OFT session to assess locomotor activity. This pre-assessment was conducted to control for individual variability and to eliminate potential locomotor confounders in the interpretation of post-treatment behavioral outcomes.

To assess the potential effects of whole-brain irradiation on locomotor activity and to evaluate whether niacin modulates this outcome, the post-treatment OFT was conducted on Day 22 of the experiment. During this session, each rat was placed in the center of an open field arena (50 × 50 × 40 cm) and observed for 5 min. Locomotor activity was quantified by counting the total number of ambulations, defined as the number of floor segments crossed with all four paws. Behavior was recorded using an external camera, and the arena was cleaned and dried between sessions to eliminate olfactory cues.

### 4.5. Passive Avoidance Learning (PAL)

Learning and memory performance of the pups was assessed with the PAL test described previously [61,62,63]. The PAL behavioral test is a test.

The PAL box consisted of two equal-area (20 × 20 × 20 cm) dark and light rooms. The floor of the dark room could be electrified using a shock generator. For the transition from one room to the other, there was a small rectangular square that could be closed with an opaque door. Normally, rats tend to move from the bright room to the dark room. First, the rat was placed in the bright room for 10 s, then the door was opened, and when it moved into the dark room, the door was closed and a 1.5 mA electric shock was given immediately for 3 s. The rat was then placed in a dark chamber. 24 h later, the rats were placed in the PAL box again. Then, 24 h later, the procedure was repeated without shock, and the rat was considered to have entered the dark area when it entered the area with its four paws. Rat was placed in a light room, and the transition time to the dark room was calculated. This period was called the latent period and was recorded for a maximum of 5 min [64].

### 4.6. Hippocampus and Cerebellum Histopathology

Cornu Ammonis (CA) 1 and CA 3 regions of the hippocampus and cerebellum were assessed for neuronal damage. Briefly, rat brains were removed after behavioral testing and fixed in 0.1 M phosphate-buffered saline (PBS) (10% formaldehyde), after which they were transferred into 30% sucrose at 4 °C. Coronal brain sections (40 µm) were prepared and fixed on gelatinized glass slides. Brain tissue sections were incubated in 10% hydrogen peroxide (H_2_O_2_) for 30 min to inhibit endogenous peroxidase activity for GFAP staining. They were then blocked with 10% goat serum (Invitrogen) for 1 h at room temperature. Primary antibodies (anti-GFAP, Abcam, Cambridge, UK; 1:1000) were applied and incubated at 4 °C for 24 h. Histostain-Plus kit (Invitrogen, Waltham, MA, USA) with DAB as chromogen was used to detect. After PBS washing, images were taken with an Olympus (Tokyo, Japan) BX51 microscope and C5050 camera [64]. GFAP-positive cells were captured at 40× magnification in 3–4 random sections per rat.

Cresyl violet, which gives a purple-blue appearance, was applied to stain Nissl bodies in neurons. Image analysis system counted survival neurons in 6 sections from each group.

#### Neuronal Counting Procedure in Hippocampus

Neuronal counting was performed in the dorsal hippocampal region using coronal brain sections corresponding to stereotaxic coordinates ranging from –2.80 mm to –3.60 mm from bregma, according to the Paxinos and Watson rat brain atlas. This specific range was chosen to ensure consistent rostro-caudal sampling and to minimize variability in neuronal density along the hippocampal axis.

To standardize the analysis, three non-overlapping coronal sections were obtained from each animal within the defined coordinate range. These sections were selected at comparable rostro-caudal levels across all animals. The mean value of the neuronal counts from these sections was used for statistical evaluation. All brain sections were collected, processed, and analyzed in a blinded manner to eliminate observer bias.

Figure 5 illustrates representative images from the CA1–CA3 regions at both low (×4) and high (×40) magnifications, highlighting the anatomical consistency of the sampled hippocampal areas across experimental groups.

### 4.7. Brain Biochemical Analysis

Brains were removed and frozen at 20 °C. Whole brain tissues were homogenized in PBS (pH 7.4) and centrifuged at 5000× *g* for 15 min. Bradford assay using BSA determined protein concentrations in the supernatants.

BDNF, CREB, TNF-α, SIRT1, and SIRT6 levels were measured using commercially available rat-specific ELISA kits. Specifically, BDNF was measured using Abcam’s Rat BDNF ELISA Kit (Cat# ab213899), CREB was measured using MyBioSource’s Rat CREB ELISA Kit (MBS2504589) (San Diego, CA, USA), TNF-α using Abcam’s Rat TNF-α ELISA Kit (Cat# ab100785), SIRT1 using MyBioSource’s Rat SIRT1 ELISA Kit (Cat# MBS2600246), and SIRT6 using MyBioSource’s Rat SIRT6 ELISA Kit (Cat# MBS063651). The intra-assay coefficients of variation (CVs) for these kits were all below 8%, ensuring reliable quantification.

### 4.8. Measurement of Brain Lipid Peroxidation (MDA)

Brain lipid peroxidation was evaluated by measuring MDA content with TBARS test. TBARS reagent and trichloroacetic acid were added to brain tissue samples and incubated at 100 °C for 60 min. Then the samples were cooled on ice. Cooled brain samples were centrifuged (3000 rpm/20 min). Supernatant absorbance was evaluated at 535 nm. Levels of MDA were determined from a standard curve and expressed as nmol/g protein.

### 4.9. Statistical Analysis

The sample size (*n* = 7 per group) was determined based on previous studies using the same whole-brain irradiation model in rats [57], in accordance with the 3R ethical principles, aiming to minimize animal use while ensuring statistically valid and reliable outcomes. Post hoc power analyses for the behavioral assessments were performed using G*Power (version 3.1.9.7).

All statistical analyses were performed using SPSS software (version 25.0; IBM, Armonk, NY, USA). All graphical representations were created using GraphPad Prism version 8.0 (GraphPad Software, San Diego, CA, USA). The Shapiro-Wilk test was employed to assess the normality of continuous data distributions. Homogeneity of variances among groups was evaluated using Levene’s test. For parameters that met the assumptions of normality and equal variances, comparisons between groups were conducted using one-way analysis of variance (ANOVA) followed by Tukey’s post hoc test. In cases where the assumption of homogeneity of variances was violated, Tamhane’s T2 post hoc test was applied. Data are presented as mean ± standard error of the mean (SEM), and a *p*-value of less than 0.05 was considered statistically significant.

## 5. Conclusions

This study demonstrates that niacin ameliorates cognitive deficits and anxiety, reduces astrocyte activation, and attenuates neuroinflammation and oxidative stress following whole-brain irradiation in rats. These effects are associated with increased expression of SIRT1 and SIRT6, leading to suppression of TNF-α and MDA levels and enhanced neuronal survival in the hippocampus and cerebellum. Niacin also promotes SIRT1-mediated activation of the CREB/BDNF pathway, which may contribute to improved behavioral and cognitive outcomes. These results highlight the neuroprotective potential of niacin, which may act by enhancing neuronal plasticity and mitigating radiation-induced brain injury. Further studies are warranted to clarify its molecular mechanisms, determine sex-specific responses, and evaluate its long-term efficacy in clinical settings.

### Limitations

One key limitation of this study is the relatively short follow-up period (~3 weeks post-WBI), which may not fully capture the delayed onset and progression of radiation-induced cognitive impairment. Future studies with longer observation windows (e.g., 8–12 weeks), including late-stage behavioral and histological evaluations, are warranted to determine the long-term durability of niacin’s neuroprotective effects.

A limitation of this study is the inclusion of only female rats, without monitoring their estrous cycle during behavioral testing. Although hormonal fluctuations across the estrous cycle could theoretically influence social behavior, accumulating evidence indicates that such effects are generally modest and do not significantly impact outcomes in three-chamber social interaction paradigms. Previous studies have shown that basic sociability and social novelty behaviors remain stable across different estrous phases [65]. Moreover, meta-analytical findings confirm that female rodents are not inherently more behaviorally variable than males [66]. Importantly, several behavioral neuroscience studies using female rats without estrous cycle monitoring have produced valid and reproducible results [67,68]. In this study, the use of sex- and age-matched experimental animals and balanced sample sizes across groups likely mitigated potential confounding effects. Nevertheless, future studies may benefit from tracking estrous stages to further enhance the interpretability of sex-specific behavioral outcomes. Given existing evidence that sex and hormonal status can influence vulnerability to radiation-induced cognitive decline, future studies should include male subjects to evaluate the generalizability of niacin’s neuroprotective effects. Given existing evidence that sex and hormonal status can influence vulnerability to radiation-induced cognitive decline, future studies should include male subjects to evaluate the generalizability of niacin’s neuroprotective effects.

## Data Availability

Data are available on request due to ethics/privacy.
